# Diagnostic challenge of the brown tumors in developing country: A case series

**DOI:** 10.1016/j.ijscr.2024.110221

**Published:** 2024-08-28

**Authors:** Erny Khomariyah, Yunita Purnamasari, Mohammad Hardian Basuki, Stepanus Massora

**Affiliations:** a Department of Orthopedics and Traumatology, Faculty of Medicine, Universitas Airlangga, Surabaya, Indonesia; b Department of Orthopedics and Traumatology, Dr. Soetomo General Academic Hospital, Surabaya, Indonesia; c Division of Nuclear Medicine, Department of Radiology, Dr. Soetomo General Academic Hospital, Surabaya, Indonesia

**Keywords:** Hyperparathyroidism, Brown tumor, Pathological fracture, Hypercalcemia, Case series

## Abstract

**Introduction and importance:**

Brown tumors are non-neoplastic reactive tissue with osteoclasts multinucleated giant cells, and vascular and proliferative fibrous tissue. Hemorrhage results in significant bone resorption caused by hyperparathyroidism. This study provides information about the diagnosis challenge for brown tumor cases.

**Case description:**

We report four cases that experience pathological fractures with moderate pain, multiple lytic lesions, severe hypercalcemia, increased ALP (Alkaline Phosphatase), and PTH (Parathyroid Hormone). Abnormal parathyroid mass was found in ultrasound. A bone scan described increasing radio uptake in various bones, a whole body scan, or *parathyroid scintigraphy* with ^99m^Tc-*MIBI*. Eventually, in all cases, the diagnosis of the brown tumor was confirmed through histopathological examination, which showed multiple nucleated giant cells with a deposit of hemosiderin pathognomonic for the brown tumor, and the diagnosis of primary hyperparathyroidism (PHPT) was confirmed in all cases.

**Clinical discussion:**

These cases show that initial misdiagnosis of brown tumors as other bone tumors. A definitive diagnosis of brown tumors requires more than just histological confirmation. A comprehensive evaluation encompassing laboratory tests, imaging studies, clinical symptoms, and a multidisciplinary orthopedic-oncology board discussion is essential. Observations show that treating hyperparathyroidism alleviates the osteolytic lesions caused by brown tumors, despite the lack of clinical guidelines.

**Conclusion:**

Based on a review of the literature and our clinical experience, we recommend screening serum calcium, phosphorus, and PTH levels in patients presenting with multiple lesions and widespread bone pain. This screening aims to rule out multiple bone lesions caused by PHPT.

## Highlights

• Misdiagnosis of multiple lytic lesions
• The diagnostic challenge of brown tumors
• MIBI scan still useful to diagnose brown tumors

## 1 Introduction and importance

Brown tumors are reactive osteolytic lesions caused by hyperparathyroidism. Although musculoskeletal manifestations in patients with primary hyperparathyroidism (PHPT) can reach 54.7 %, the incidence of brown tumors ranges from 1.5 % to 4.5 %. Misdiagnosis of brown tumors as giant cell tumors has been reported. Both have a similar appearance, as osteolytic bone lesions on imaging and as mononuclear cells with multinuclear giant cells on histopathology examination [1,2]. Diagnosis can be particularly challenging when a brown tumor is the initial presenting manifestation of hyperparathyroidism [3].

Brown tumor is generally diagnosed based on medical history, clinical examination, imaging, and laboratory results confirmed by histopathology findings. The clinical manifestations of this condition include a lump or swelling, bone pain, and pathological fracture. Imaging may reveal osteoporotic lesions, cortical bone thinning, and bone destruction in multiple localized cysts [4]. Histopathology of the brown tumor lesion typically shows non-neoplastic reactive tissue, extensive bone resorption, osteoclasts with multinucleated giant cells, vascular and proliferating fibrous tissue, and hemorrhage. While histopathology remains the reference standard, the most crucial element for the diagnosis of brown tumors is recognizing hyperparathyroidism due to extremely high levels of parathyroid hormone (PTH) and serum calcium. However, PTH is not routinely checked and can be easily overlooked especially in developing countries due to limited facilities and high examination costs. We can carry out other tests using parathyroid imaging, ultrasound, and sestamibi scintigraphy are the most commonly used imaging modalities. Accurate diagnosis is vital in order to circumvent superfluous diagnostic procedures, complications and therapeutic interventions as brown tumors may regress spontaneously with the treatment of hyperparathyroidism [3,5].

## 2 Methods

This is restrospective case series, include four cases of brown tumor caused by PHPT to provide a reference for future clinical practice reported from 2022 to 2023. All patients in this case series were treated in the Orthopedic department, for biopsy and parathyroidectomy done by the Head and Neck department of DR Soetomo General Hospital, Surabaya, and we obtained written informed consent from all patients. This case series has been reported in line with PROCESS criteria.

## 3 Results

### 3.1 Case 1 presentation

Our first patient is a woman 22 years old with a pathological fracture of the humerus and an 8-month history of intermittent pain with deformity in the shoulder, referred to as suspected Ossifying fibroma. On initial examination, the patient was suspected of having a brown tumor due to the presence of multiple lytic lesions of the humerus and bilateral femur, hypercalcemia (14.8 mg/dL) with decreased phosphate (1.74 mg/dL), an elevation of ALP, and young age without other comorbidities. The patient was subsequently brought to a multidisciplinary discussion to determine the most appropriate diagnostic pathway and to establish a diagnosis. Subsequently, several diagnostic approaches were conducted. we found an elevation of PTH (2220 mg/dL). The USG showed a cystic lesion in the thyroid, CT-scan cervical with contrast showed a cystic lesion of the left lobe of the thyroid and left parathyroid, and multiple lytic lesions were visualized in the spine, sternum, costae, clavicula, scapula, bilateral maxilla, and calvaria. *Parathyroid scintigraphy* with ^99m^Tc-*MIBI* showed parathyroid adenoma at the lower pole of the right thyroid.

The patient got a total thyroidectomy and a partial parathyroidectomy. The histopathology results indicate an adenomatous goiter along with a parathyroid adenoma. The biopsy results of the lesion in the right humerus revealed a multinucleated giant cell with vascular fibroblastic stroma and hemosiderin deposits. The patient had a good clinical and laboratory outcome, but we lost the patient's follow-up after five months (Table 1).

**Table 1:** Characteristics, laboratory, imaging, and clinicohistopathology features of patients.

No	Variable	Case 1	Case 2	Case 3	Case 4
1	Age	22 y.o.	24 y.o.	40 y.o.	26 y.o.
2	Sex	F	F	F	M
3	Pathological fracture (total site)	Right humerus, left femur	Left humerus, left femur	Right humerus, left radius et ulna, right femur	Right humerus
4	Site of multiple lytic lesions	Mandibula, maxilla, costae, humerus, bilateral femur, pelvis	Calvaria, Left humerus, bilateral tibia and femur	Calvaria, multiple costae, spine, pelvis, and long bone	Humerus, costae, radius, ulna and pelvis
5	PTH Levels Preoperative (14 to 65 pg/m)	2220 pg/mL	415 pg/mL	No data	740 pg/mL
6	PTH levels postoperative (14 to 65 pg/m)	No data	70.5 pg/mL	No data	No data
7	TSH pre operative (0.5–5.0 mIU/L)	0,937 mIU/L	No data	0.503 mIU/L	0.812 mIU/L
8	FT 4 pre-operative (0.8–1.9 ng/L)	0.73 ng/dL	No data	0.99 ng/dL	8.2 ng/dL
10	Calcium levels preoperative (8.5 to 10.2 mg/dL)	14.89 mg/dL	13.1 mg/dL	18.4 mg/dL	14.7 mg/dL
11	Calcium postoperative (8.5 to 10.2 mg/dL)	8.7 mg/dL	8.7 mg/dL	9.2 mg/dL	8.5 mg/dL
12	Phosphate levels preoperative (2.5 to 4.5 mg/dL)	2.1 mg/dL	2.12 mg/dL	2.35 mg/dL	2.06 mg/dL
13	Phosphate levels postoperative (2.5 to 4.5 mg/dL)	2.89 mg/dL	2.98 mg/dL	2.7 mg/dL	2.95 mg/dL
14	BUN (6 to 24 mg/dL)	7.1 mg/dL	9.3 mg/dL	24.3 mg/dL	12.9 mg/dL
15	Creatinine serum (0.7 to 1.3 mg/dL)	0.5 mg/dL	0.6 mg/dL	1.1 mg/dL	1.9 mg/dL
16	ALP levels preoperative (40–100 U/L)	1990 U/L	872 U/L	716 U/L	431 U/L
17	Vitamin D (20 ng/mL–50 ng/mL)	7.6 ng/mL	15.6 ng/mL	No data	9.6 ng/mL
18	A whole body scan, or *parathyroid scintigraphy* with ^99m^Tc-*MIBI*	Parathyroid adenoma at the lower pole of the right thyroid	The process of suspected malignant metastasis in the left scapula, bilateral lungs, and proximal 1/3 of the left femur.	High radioactive uptake on the projection of the right thyroid and a slight decrease in the 2-hour image suggest a disorder in the right thyroid or a parathyroid disturbance.	A large parathyroid adenoma is located at the level of the left thyroid lobe.
19	Ultrasonography	Benign cystic of inferior lobes of the thyroid	A mass in the left infra-thyroid region suggests a suspicion of a left parathyroid mass	A solid hypoechoic lesion in the posterior aspect of the right thyroid lobe is suspicious for a parathyroid mass.	Solid mass in the left lobe
20	CT scan cervical contrast	showed a cystic lesion at the left inferior thyroid	Massa solid hypodense	Enhancing solid mass ± 1,0 × 1,8 × 3,6 cm in the posterior lobe on the right thyroid	No data
21	Histo-pathological examination	Parathyroid adenoma and adenomatous goiter with hyperplastic nodule	Atypical parathyroid tumor (borderline malignancy), adenomatous goiter	Papillary thyroid carcinoma – microcarcinoma, upper right parathyroid adenoma	Parathyroid adenoma
22	Operative procedure	Parathyroidectomy and total thyroidectomy, ORIF for left femur and Conservative for right humerus	Parathyroidectomy superior sinistra + lobectomy sinistra, ORIF for left femur, and conservative for left humerus	ORIF plating femur + biopsy + parathyroidectomy	Conservative treatment with splinting

### 3.2 Case 2 presentation

The 24-year-old woman presented with a pathological fracture in the femur with a history of a solid lump and moderate pain in the left knee for 5 months. A biopsy done before being referred to us showed low-grade proximal femur fibrosarcoma. Laboratory tests showed an elevation in calcium (13.1 mg/dL) with decreased phosphate (2.12 mg/dL) and PTH (415 mg/dL). X-rays showed multiple lytic lesions in the humerus, femur, and tibia (Fig. 2A). The examination using a whole body scan with ^99m^Tc-*MIBI* showed pathological uptake of radiopharmaceuticals in the projections of the left scapula, on both sides of the costae, and in the proximal 1/3 of the left femur and unable to determine the location of the primary malignancy. An ultrasonography revealed a mass in the left infra-thyroid, indicating a possible left parathyroid mass.

A thorax CT scan revealed multiple osteolytic lesions in the bilateral ribs and left scapula, accompanied by a soft tissue mass around them. A cervical CT scan with contrast revealed a solid mass in the posteroinferior aspect of the left thyroid lobe, supporting the parathyroid mass. The bone mass density (BMD) examination revealed a T-score value of −3.7, indicating osteoporosis. A parathyroidectomy was performed on the patient. The histopathology was examined in the parathyroid and the thyroid showed an atypical parathyroid and adenomatous goiter and the tibia showed brown tumor. The X-ray evaluation 6 months post-operation revealed an improvement in lytic lesions. Post-operation, PTH values decreased from 415 pg/mL to 70.9 pg/mL, and calcium levels decreased from 13.1 mg/dL to 8.4 mg/dL (Table 1).

### 3.3 Case 3 presentation

A 40-year-old woman with pathological fractures in the left shoulder, arm, forearm, and femur suspected multiple myeloma. Laboratory tests revealed hypercalcemia (18.4), with elevated ALP to 716, LDH to 257, and elevated RFT. The X-ray examination found multiple lytic lesions at the humerus, radius, ulna, and femur with osteopenia. We did another diagnostic ultrasound, which showed a parathyroid mass. The whole-body scan with ^99m^Tc-*MIBI* showed high uptake in the right thyroid projection, suggesting right thyroid or right parathyroid disease. We did not perform a parathyroid scan with ^99m^Tc-*MIBI* at that time.

In the histopathological examination, papillary thyroid carcinoma with parathyroid adenoma. Our patient had undergone a right isthmus-lobectomy and a right parathyroidectomy superior and inferior, with preservation of the right recurrent laryngeal nerve. There was a decrease in calcium levels and improved renal function, as well as clinically good outcome after surgery (Table 1).

### 3.4 Case 4 presentation

A 26-year-old male presented with a pathological fracture of the right humerus, suspected simple bone cyst, and differential diagnosis of fibrous dysplasia. X-ray examination showed a fracture of the right humerus and multiple lytic lesions at the radius, ulna, proximal femur, and pelvis (Fig. 4A). Laboratory findings revealed elevated levels of calcium (14.3 mg/dL), phosphorus (2.06 mg/dL), alkaline phosphatase (ALP) (431 U/L), and elevated PTH (740 mg/dL) (Table 1).

A bone scan showed diffusely increased radioactivity in the calvaria, mandible, left scapula, ribs, right humerus, bilateral proximal femurs, and bilateral tibias, consistent with a metabolic bone disease caused by hyperthyroidism. *Parathyroid scintigraphy* with ^99m^Tc-*MIBI* showed a large parathyroid adenoma located at the level of the left thyroid lobe. Ultrasounds showed a parathyroid mass. A partial parathyroidectomy has been performed, with histopathology results confirming parathyroid adenoma (Table 1).

This case highlights the complex management considerations for pathological fractures associated with bone cysts, necessitating integrated therapeutic approaches encompassing orthopedic intervention and metabolic management.

## 4 Discussion

The parathyroid glands secrete parathyroid hormone (PTH), which regulates calcium and phosphorus metabolism. PTH plays an important role in tooth development and mineralization and increases bone resorption. Primary HPT (PHPT) is an endocrinopathy condition characterized by hypersecretion of PTH, which may be caused by an adenoma (solitary or multiple), idiopathic hyperplasia, or parathyroid carcinoma. This condition induces excessive secretion of parathyroid hormone (PTH). In hyperparathyroidism (HPT) cases, the overproduction of parathormone (PTH) may affect any part of the skeleton, potentially leading to the formation of single or multiple cyst-like lesions of bone, known as brown tumors. These are thought to be caused by rapid osteoclastic activity and peri-trabecular fibrosis, which can result in erosive bony lesions. These form a type of local destructive phenomenon due to HPT. They appear to represent a reparative cellular process rather than true neoplasia.

Brown tumor symptoms are often atypical, manifesting as pain, edema, gastrointestinal, neurological complaints, and occasionally pathologic fractures in the local area. Due to a high level of parathyroid hormone-related peptide (PTHrp), which mimics the action of PTH, malignant tumors can also cause similar symptoms as part of the neoplastic syndrome. As a result, misdiagnosis rates are high, especially when surgeons are inexperienced in recognizing brown tumors. Many clinical techniques, such as imaging studies, pathological evaluation, laboratory testing, and clinical examination, are used to improve diagnostic accuracy [5].

**Fig. 1:**
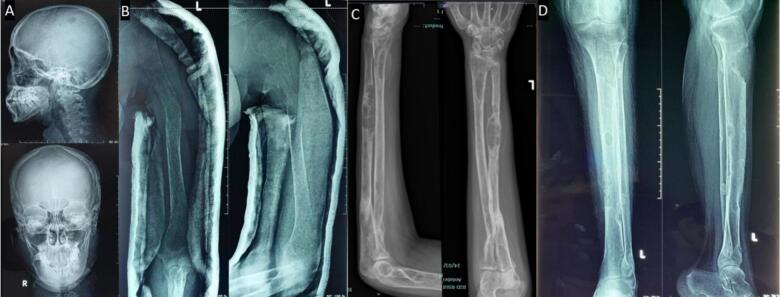
X-ray radiograph of our patient with brown tumor, showing multiple osteolytic lesions with cortical thinning in the skull (A), humerus (B), radius, ulna (C), and tibia (D) characteristic of hyperparathyroidism.

**Fig. 2:**
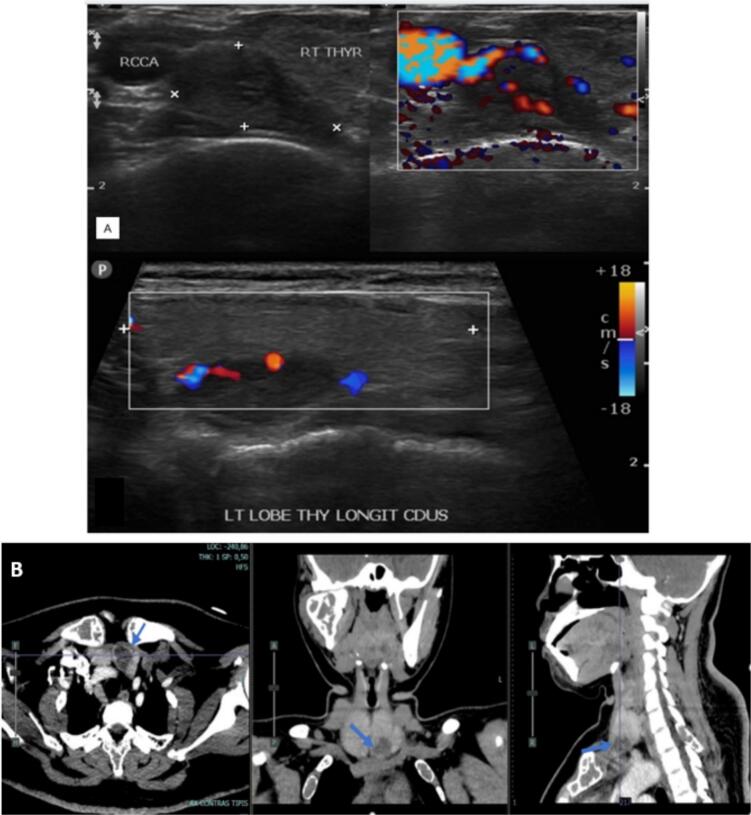
Ultrasound image of a patient with brown tumor, showing a hypoechoic lesion in the parathyroid gland consistent with the diagnosis (A), cervical CT scan with contrast revealed a solid mass in the posteroinferior aspect of the left thyroid lobe, supporting the parathyroid mass (B).

**Fig. 3:**
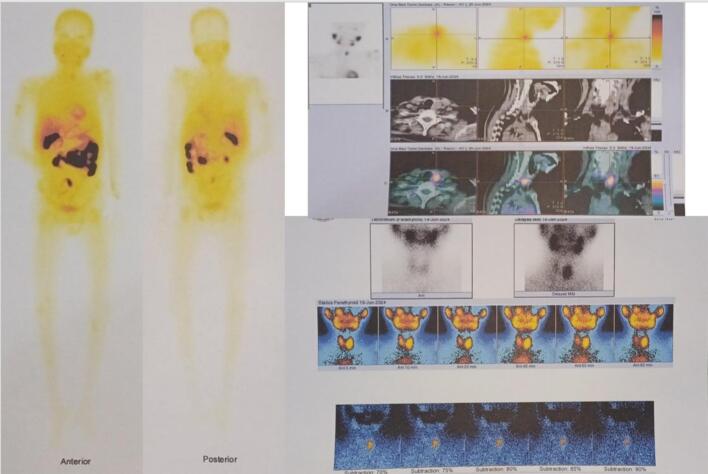
The examination using a whole body scan with ^99m^Tc-*MIBI* showed pathological uptake of radiopharmaceuticals in the projections in multiple sites and *parathyroid scintigraphy* with ^99m^Tc-*MIBI* showed a large parathyroid adenoma located at the level of the left thyroid lobe.

**Fig. 4:**
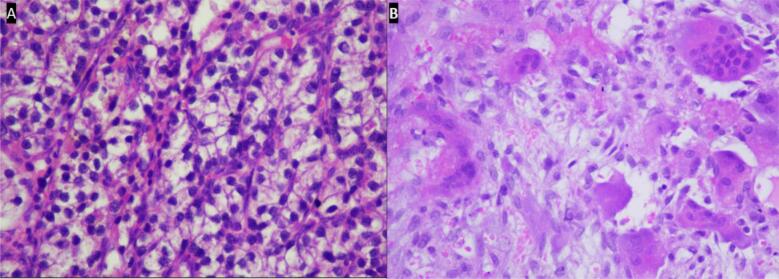
The biopsy of parathyroid showed results adenoma parathyroid (A), biopsy of the lesion in the cruris revealed a multinucleated giant cell with vascular fibroblastic stroma and hemosiderin deposits.

We present cases of previously misdiagnosed brown tumors from PHPT as other bone diseases. The diagnosis of brown tumors cannot be made based on histological findings alone. It also requires a comprehensive investigation that includes discussion with the multidisciplinary orthopedic oncology board consisting of Orthopedic Oncology, Radiologist, Internist, Nuclear Medicine Specialist, Head and Neck surgeon, and Pathologist based on imaging, laboratory tests, and clinical complaints. Our cases presented with pathological fractures, moderate pain, multiple lytic lesions (Fig. 1), severe hypercalcaemia, increased ALP (alkaline phosphatase) and PTH (parathyroid hormone). An ultrasound examination revealed the presence of an unusual mass in the parathyroid region and Cervical CT scan with contrast revealed a solid mass in the posteroinferior aspect of the left thyroid lobe, supporting the parathyroid mass (Fig. 2). A bone scan indicated an increase in radio uptake in various bones, which was further investigated with a whole body scan or parathyroid scintigraphy with 99mTc-MIBI (Fig. 3). Ultimately, in all cases, the diagnosis of the brown tumor was confirmed through histopathological examination, which showed multiple nucleated giant cells with a deposit of hemosiderin, which is pathognomonic for the brown tumor (Fig. 4). In addition, the diagnosis of primary hyperparathyroidism (PHPT) was confirmed in all cases.

Due to the lack of routine screening of serum calcium and parathyroid hormone in developing countries, fracture is the first sign suggesting the disease our cases are an example. Multiple osteolytic lesions at the fracture site are frequently mistaken for malignant tumors such as multiple myeloma, osteosarcoma, Ewing sarcoma, lymphoma, and metastatic carcinoma. The radiological difference in our cases was that the brown tumor did not invade adjacent tissues or induce changes around the periosteum. In contrast, a periosteal reaction is frequently observed in cases of primary or metastatic tumors [5].

In the presence of systemic osteolytic bone loss with severe osteoporosis, hypercalcemia, and elevated alkaline phosphatase levels, it is extremely important to discuss the likelihood of bone metabolic diseases, particularly brown tumors induced by hyperparathyroidism. Partial or total removal of the parathyroid glands is the standard treatment for brown tumors [6].

Our patient is much younger than average, supporting the findings of Al-Gahtany et al., who reviewed 16 cases of brown tumors at the skull base and revealed that the average age of the patients was 32 years, with 75 % of them being women [7]. Resendiz-Colosia et al. reported a series of 22 cases of maxillofacial brown tumors, showing that 91 % of them were women [8]. One possible explanation is that young women have a higher susceptibility to PTH and a predisposition to brown tumors. However, the exact causes still need to be determined [9].

In our case series, the average preoperative calcium level was 15.27 ± 2.23 mg/dL, and the average preoperative PTH level was 1125 ± 962.12 pg/mL. These findings are consistent with Kartini et al., who explained that calcium levels greater than 14 mg/dL and PTH levels elevated 3–10 times above normal indicate the presence of a parathyroid tumor. This alignment underscores the diagnostic relevance of elevated calcium and PTH levels in identifying parathyroid tumors among our patients.

A multidisciplinary strategy is necessary for the proper treatment of pathological fractures caused by brown tumors. This approach should be directed towards managing parathyroid tumors and achieving an optimal bone union of pathological fractures and osteolytic lesions. The main goals of treatment were to manage levels of calcium, phosphate, and hyperparathyroidism. For certain conditions, parathyroidectomy is a suitable treatment, and following this procedure, brown tumors often regress. Stabilizing the bone microenvironment through the normalization of serum calcium levels is the main objective. It is also advised to use bisphosphonates for therapy [10,11].

A whole-body scan with ^99m^Tc-*MIBI* is a valuable tool for assessing brown tumors that develop due to primary hyperparathyroidism (PH). However, it may not always detect small brown tumors that are visible on whole-body bone scans. Parathyroid hormone (PTH) levels have been identified as optimal for diagnostic accuracy and predicting positive outcomes in both ^99m^Tc-sestamibi scans and bone scans. On the other hand, alkaline phosphatase (ALP) levels have proven useful for predicting a negative imaging outcome. These findings highlight the complementary roles of PTH and ALP in guiding the diagnostic and prognostic evaluation of brown tumors associated with primary hyperparathyroidism [12].

**Fig. 5:**
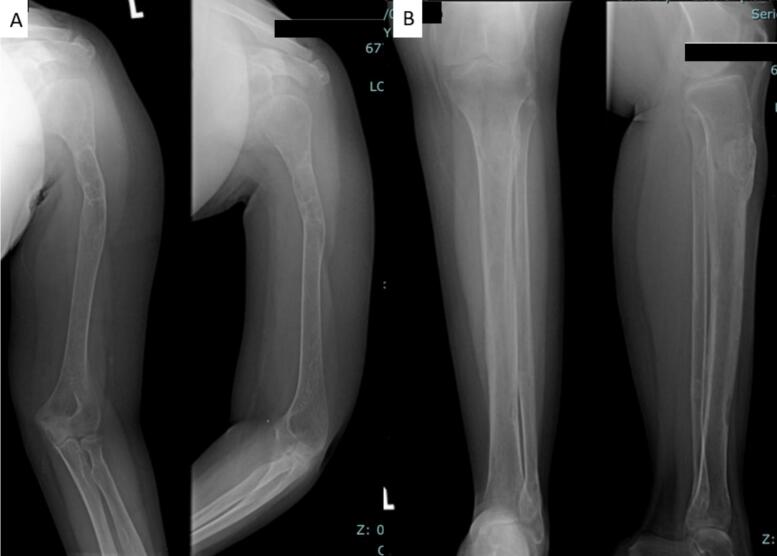
X-ray radiograph follow up 6 months post treatment hyperparathyroidism (parathyroidectomy), callus formation and thicker cortical showed regressed of brown tumor.

After parathyroidectomy, a medical condition known as “hungry bone syndrome” may arise, which is characterized by rapid and sustained hypocalcemia. Determinate orthopedic surgery should be postponed until calcium control is accomplished. Conversely, there have been reports that preferable orthopedic fixation was done before parathyroidectomy. Which lesions are most likely to need orthopedic surgery is a matter of debate. The Mirrel criteria-like objective grading system is not available to help us choose the best course of action for treating brown tumors. As a result of pathological fractures at the lower extremity, all of the patients in our case series had orthopedic surgery performed before parathyroidectomy. Splinting and vitamin D and calcium supplements, however, were the conservative treatments we used for another lytic lesion. Clinically, in labs and radiology, every patient gets better (Fig. 5) (Table 1). All patients get better clinically, in laboratory and radiology, but we need longer observation time to evaluate the outcome.

Measuring PTH levels and performing biochemical studies are essential for diagnosis. The treatment algorithm is not widely agreed upon. The location, size, and degree of pain all help determine the best course of treatment. Orthopedic operations are necessary for certain lesions, while parathyroidectomy is adequate for others. Before surgery, consultations in general surgery, endocrinology, and anaesthesiology are crucial. A comprehensive strategy is necessary for the treatment of brown tumors.

**Fig. 6:**
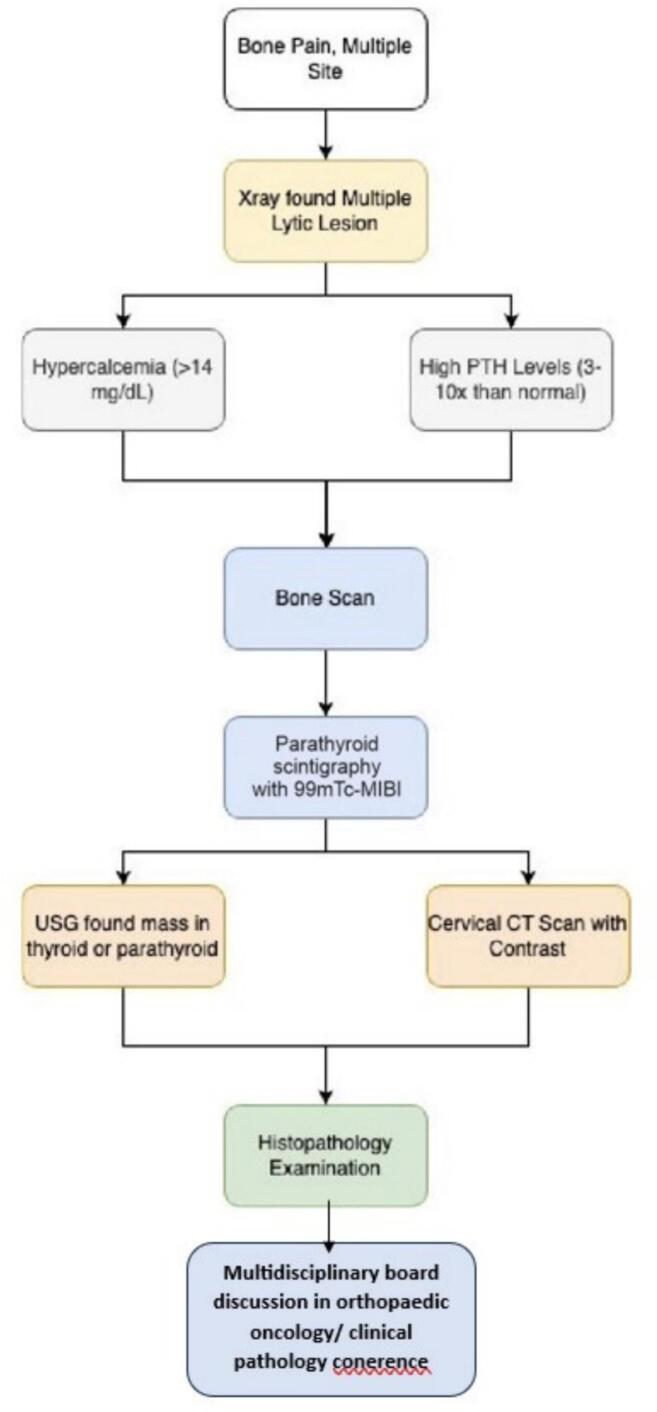
The algorithm of diagnosis steps in our cases.

Diagnostic steps must be carried out systematically and sequentially to determine the diagnosis and potential complications in cases of brown tumors. In this report, the diagnostic approach was conducted in a step-by-step manner (Fig. 6). We want to emphasize the importance of considering PHPT in the differential diagnosis of patients with multiple lytic bone lesions, thereby avoiding unnecessary and harmful interventions. This case series has been reported following the PROCESS 2023 Criteria [13].

The present study is limited by its retrospective design, short evaluation period, and incomplete follow-up due to patient-related factors such as distance and cost constraints. A larger, prospective study with a longer follow-up period would undoubtedly provide more insight. However, this paper presents a valuable case series on brown tumors at a single institute, offering crucial insights into the diagnosis.

## 5 Conclusion

Based on our case series, we recommended that screening serum calcium, phosphorus, and parathyroid hormone (PTH) examinations or *Parathyroid scintigraphy* with ^99m^Tc-*MIBI*, and ultrasound be performed for patients presenting with multiple lesions and multiple sites of bone pain. This conclusion was reached following a review of the relevant literature and a detailed reflection on the diagnostic and treatment processes employed in our case series. The purpose of this screening is to exclude multiple bone lesions from PHPT.

## Informed consent

Appropriate consent was obtained from all individual participants included in the study.

## Ethical approval

Regarding to the observational study of outcome in our case series, the ethical approval was waived by our institution. However, the copies of informed consent are available for review by the Editor-in-Chief of this journal on request.

## Funding

None.

## Guarantor

Mohammad Hardian Basuki.

## CRediT authorship contribution statement

Erny Khomariyah involved in conceptualization, data curation, formal analysis, investigation, methodology, visualization, project administration, writing- original draft.
Yunita Purnamasari involved in conceptualization, data curation, formal analysis, investigation, methodology, visualization, project administration, writing- original draft.
Mohammad Hardian Basuki involved in conceptualization, data curation, formal analysis, investigation, methodology, visualization, validation, project administration, writing-review editing.
Stepanus Massora involved in conceptualization, data curation, formal analysis, writing-review editing.

## Declaration of competing interest

The authors have no conflicts of interest to disclose.
